# Characteristics of glioblastomas and immune microenvironment in a Chinese family with Lynch syndrome and concurrent porokeratosis

**DOI:** 10.3389/fonc.2023.1194232

**Published:** 2023-07-17

**Authors:** Zhi-Gang Yao, Fang Hua, Zuo-Hua Yin, Ying-Jie Xue, Yang-Hao Hou, Yi-Cong Nie, Zhi-Ming Zheng, Miao-Qing Zhao, Xiao-Hong Guo, Chao Ma, Xiao-Kang Li, Zhou Wang, Guang-Cun Liu, Gui-Hui Zhang

**Affiliations:** ^1^ Department of Pathology, Shandong Provincial Hospital Affiliated to Shandong First Medical University, Jinan, Shandong, China; ^2^ Department of Pathology, Shandong Provincial Hospital, Cheeloo College of Medicine, Shandong University, Jinan, Shandong, China; ^3^ Department of Microbiology and Immunology, Tulane University, New Orleans, LA, United States; ^4^ Department of Pathology, The People’s Hospital of Huaiyin, Jinan, Shandong, China; ^5^ Department of Pathology, Chongqing Medical University, Chongqing, China; ^6^ Department of Neurosurgery, Shandong Provincial Hospital Affiliated to Shandong First Medical University, Jinan, Shandong, China; ^7^ Department of Pathology, Shandong Cancer Hospital and Institute, Shandong First Medical University and Shandong Academy of Medical Sciences, Jinan, Shandong, China; ^8^ Department of Pathology, The First Affiliated Hospital of Shandong First Medical University and Shandong Provincial Qianfoshan Hospital, Jinan, Shandong, China; ^9^ Department of Dermatology, Shandong Provincial Hospital Affiliated to Shandong First Medical University, Jinan, Shandong, China; ^10^ Department of Neurosurgery, The First Affiliated Hospital of Shandong First Medical University and Shandong Provincial Qianfoshan Hospital, Jinan, Shandong, China

**Keywords:** glioblastoma, Lynch syndrome, porokeratosis, DNA mismatch repair, PD-L1

## Abstract

**Background:**

Lynch syndrome (LS)-associated glioblastoma (GBM) is rare in clinical practice, and simultaneous occurrence with cutaneous porokeratosis is even rarer. In this study, we analyzed the clinicopathological and genetic characteristics of LS-associated GBMs and concurrent porokeratosis, as well as evaluated the tumor immune microenvironment (TIME) of LS-associated GBMs.

**Methods:**

Immunohistochemical staining was used to confirm the histopathological diagnosis, assess MMR and PD-1/PD-L1 status, and identify immune cell subsets. FISH was used to detect amplification of EGFR and PDGFRA, and deletion of 1p/19q and CDKN2A. Targeted NGS assay analyzed somatic variants, MSI, and TMB status, while whole-exome sequencing and Sanger sequencing were carried out to analyze the germline mutations.

**Results:**

In the LS family, three members (I:1, II:1 and II:4) were affected by GBM. GBMs with loss of MSH2 and MSH6 expression displayed giant and multinucleated bizarre cells, along with mutations in *ARID1A*, *TP53*, *ATM*, and *NF1* genes. All GBMs had TMB-H but not MSI-H. CD8+ T cells and CD163+ macrophages were abundant in each GBM tissue. The primary and recurrent GBMs of II:1 showed mesenchymal characteristics with high PD-L1 expression. The family members harbored a novel heterozygous germline mutation in *MSH2* and *FDPS* genes, confirming the diagnosis of LS and disseminated superficial actinic porokeratosis.

**Conclusion:**

LS-associated GBM exhibits heterogeneity in clinicopathologic and molecular genetic features, as well as a suppressive TIME. The presence of MMR deficiency and TMB-H may serve as predictive factors for the response to immune checkpoint inhibitor therapy in GBMs. The identification of LS-associated GBM can provide significant benefits to both patients and their family members, including accurate diagnosis, genetic counseling, and appropriate screening or surveillance protocols. Our study serves as a reminder to clinicians and pathologists to consider the possibility of concurrent genetic syndromes in individuals or families.

## Introduction

Lynch syndrome (LS) is an autosomal dominant condition caused by a germline heterozygous pathogenic variant in one of four DNA mismatch repair (MMR) genes (*MLH1*, *MSH2*, *MSH6*, or *PMS2*) or deletions in *EPCAM* (1). LS is associated with higher lifetime risk for colorectal cancer (CRC) and various other cancers, such as those affecting the endometrium, stomach, urinary tract, biliary tract, brain (usually glioblastoma), and skin (including sebaceous adenomas, sebaceous carcinomas, and keratoacanthomas). In the 2021 fifth edition of the World Health Organization (WHO) classification of central nervous system tumors, nineteen brain tumor predisposition syndromes (BTPSs) were described, but LS was not included (2). LS-associated glioblastoma (GBM) is rare in clinical practice. Although the histological characteristics of LS-associated GBM have been reported (3), the genetic characteristics and immune microenvironment have not been fully studied. In this study, we analyzed the clinicopathological and genetic characteristics and immune microenvironment of GBMs that occurred in three members of one family with LS. We developed a strategy to distinguish LS-associated GBM, which will benefit patients and family members for precise diagnosis, genetic counseling, as well as screening or surveillance protocols. Interestingly, the family members also had concurrent cutaneous disseminated superficial actinic porokeratosis (DSAP), which is an autosomal dominant inherited disorder that causes dry, scaly patches on the skin of arms and legs (4). Although many BTPSs often include cutaneous manifestations, such as neurofibromatosis type 1 (NF1), melanoma-astrocytoma syndrome, and basal cell nevus syndrome (2), our cases highlight the necessity of a thorough examination of dermatological conditions. It may represent an independent inherited disorder rather than a part of BTPS phenotype.

## Materials and methods

### Samples collection

We obtained tumor samples from three GBM patients who underwent surgical treatment at different hospitals, including the Shandong Provincial Hospital Affiliated to Shandong First Medical University (II:1 and II:1r), The First Affiliated Hospital of Shandong First Medical University (II:4), and Shandong Cancer Hospital and Institute (I:1). We also collected peripheral blood samples from family members for whole-genome sequencing and Sanger sequencing.

### Immunohistochemical analysis

Immunohistochemical (IHC) staining was used to confirm the histopathological diagnosis, assess MMR and PD-1/PD-L1 status, and identify immune cell subsets in the tumor microenvironment. The antibodies utilized in this study are listed in **Supplemental Table 1**. The standard avidin-biotin-peroxidase method was used to carry out the immunostaining with a Ventana BenchMark Ultra system (Roche Diagnostics). For PD-L1 IHC 22C3 staining, the Dako Autostainer Link 48 platform (Dako, USA) was used. Positive external controls were utilized in each staining process using tonsil tissue for 22C3 and placenta for SP263. The PD-L1 tumor proportion score (TPS) is determined as the percentage of PD-L1 positive stained tumor cells (TCs) with at least partial membrane staining relative to the total number of TCs, excluding tumor-associated interstitial cells (ICs), necrotic, normal or non-neoplastic cells from the evaluation. Tumor PD-L1 expression was classified into three clinically relevant TPS groups: <1% (no expression), 1-49% (low expression), and ≥50% (high expression). The evaluation of MMR immunostaining employed lymphocytes and stromal cells in the background as a positive internal control. Beforehand, negative controls for each IHC were examined to consider factors of background staining.

### O6-methylguanine-DNA methyltransferase methylation analysis

500 ng of DNA extracted from FFPE tissues was modified by sodium bisulfite, which converts unmethylated cytosine to uracil, following manufacturer’s instructions of MGMT plus Kit (Gene Tech, Shanghai, China). PCR was performed on the modified DNA and the products were sequenced. The average methylation of the 10 CpG sites was calculated using the PyroMark Q96 ID system (Qiagen). An analytical cut-off of 5% was used to distinguish methylated from unmethylated samples, as suggested by the MGMT plus Kit.

### TERT promoter mutation analysis

The 163-bp fragment was amplified using KAPA2G Robust HotStart ReadyMix (Sigma) with the primers 5’-GTCCTGCCCCTTCACCTT-3’ and 5’-CAGCGCTGCCTGAAACTC-3’. The purified PCR products were sequenced by GATC Biotech (Cologne, Germany), and the chromatograms were manually interpreted using 4Peaks Software (version 1.7.1., Mekentosj).

### Fluorescence in situ hybridization analysis

EGFR and PDGFRA amplification and 1p/19q and CDKN2A deletion were identified using FISH. The FISH assay was performed using locus-specific probes for EGFR (7p12), 1p36, 19q13, PDGFRA (4q12), CDKN2A (9p21), and corresponding reference centromeric probes recommended by the manufacturer (Agilent, Beijing, China), following previously described method (5).

### Genomic analysis of somatic and germline variants

A next-generation sequencing (NGS) assay that targets 550 somatic genes, as well as microsatellite instability (MSI) and tumor mutation burden (TMB) status, was performed at Di’An Diagnostics (Hangzhou, China) using an Illumina Hiseq4000 platform (Illumina, San Diego, CA, USA). Whole-exome sequencing (WES) was performed on DNA samples obtained from peripheral leucocytes of family members. The validation of germline mutations was analyzed using Sanger sequencing. Genomic DNA isolation, sequencing and data analysis procedures were described in previous studies (5, 6).

### DNA methylation profiling analysis

DNA was extracted from tumors and subjected to genome-wide DNA methylation analysis using Illumina Human MethylationEPIC BeadChip (850k) assay (Guangzhou Huayin Medical Laboratory Center, Guangzhou, China). Data processing and quality checks were performed using the GenomeStudio Methylation Module. To evaluate subgroups, hierarchical clustering, t-distributed stochastic neighbor embedding (tSNE), and principal component analysis were conducted using the R package Rtsne, and pheatmap was used with the WPGMA linkage method and Euclidean distance (7).

## Results

### Clinical courses

#### Patient II:1

**Figure 1:**
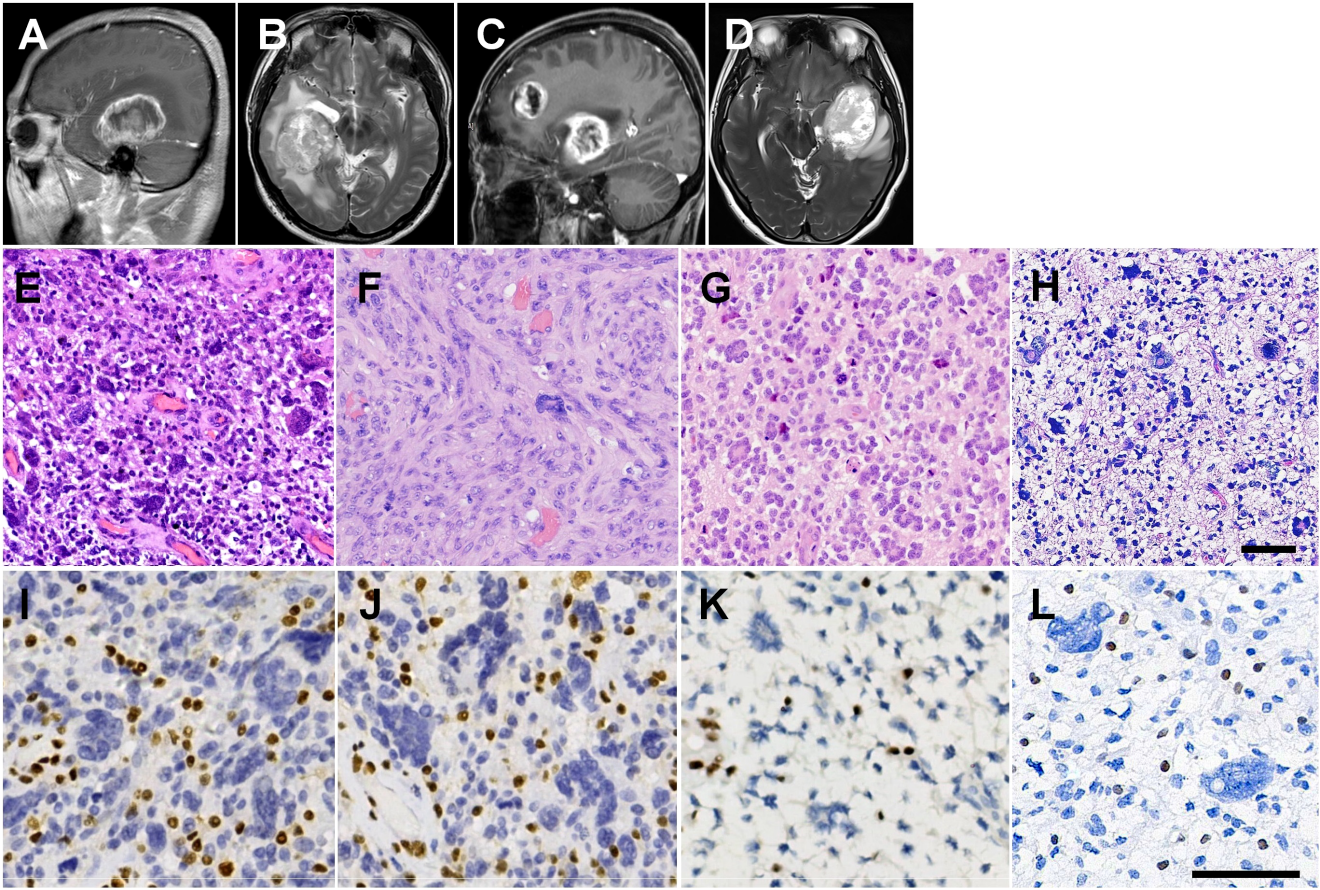
Radiological and histopathological features. **(A)** Right parasagittal enhanced T1WI image of II:1, showing a nodular mass in the right temporal lobe with ring-enhancing. **(B)** Axial heterogeneous T2WI image. **(C)** Left parasagittal enhanced T1WI image of II:4, showing two masses in the left temporal lobe and left frontal lobe with rim enhancement. **(D)** Axial heterogeneous T2WI image. **(E)** H&E staining showing many multinucleated giant cells with vascular proliferation in the primary tumor from II:1. **(F)** The recurrent GBM of II:1 showing diffuse spindle cells arranged in fascicular pattern with scattered pleomorphic cells. **(G)** Many wreath-shaped multinucleated giant cells and smaller oligodendrocyte-like cells in the tumor of II:4. **(H)** Highly pleomorphic neoplastic cells with predominance bizarre and multinucleated giant cells in the tumor of I:1. **(I)** IHC staining showing loss of nuclear staining of MSH2 in GBM of II:4. **(J)** Loss of nuclear staining of MSH6 in GBM of II:4. **(K)** IHC staining showing loss of nuclear staining of MSH2 in GBM of II:4. **(L)** IHC staining showing loss of nuclear staining of MSH2 in GBM of I:1. Scale bars = 50 μm.

The proband, a 53-year-old male, presented with intermittent frontoparietal headache for a month, without nausea or vomiting. Magnetic resonance imaging (MRI) revealed a well-defined nodular mass, measuring 5.4×5.0×5.0 cm in the right temporal lobe, with hyperintense T1 weighted image (WI) and a typical ring-enhancing pattern (**Figure 1A**), as well as heterogeneous T2WI (**Figure 1B**). Subsequently, he underwent the first surgical resection on May 7, 2018, and the pathological diagnosis confirmed glioblastoma with MMR deficiency. Two months later, MRI examination showed tumor recurrence, for which the patient received radiotherapy (DT 64 Gy, 2 Gy/day, 32 day) with concomitant daily temozolomide (TMZ, 75 mg/m^2^/day). The patient then underwent six cycles of adjuvant TMZ (cycle 1: 150 mg/m^2^/day, day1-day5, 4w; cycle 2-6: 200 mg/m^2^/day, day1-day5, q4w). After completing chemotherapy, MRI examination revealed no signs of recurrence at the surgical site. However, at 5 months after the completion of chemotherapy (1 year after the first surgery), the patient underwent MRI due to worsening headaches and cognitive impairment, which showed the presence of abnormal enhancement shadows in the original surgical area, indicating tumor recurrence. On June 18, 2019, he underwent the second intracranial tumor resection (II:1r). Two months after the second surgery, imaging examination showed recurrence once again. The patient declined PD-1 targeted immunotherapy and radiotherapy and received only TMZ treatment (200 mg/m^2^/day),. However, due to aggravated headaches and vomiting, the patient discontinued TMZ after two cycles treatment and passed away on November 26, 2019. Additionally, he underwent resection for colonic adenocarcinoma at the age of 35 years and penile squamous cell carcinoma (SCC) at the with loss of MSH2 and MSH6 expression (**Supplementary Figure 1**).

#### Patient II:4

The proband’s younger sister, aged 48 years, showing cognition and language disorders with masses in the left temporal and frontal lobes, measuring 5.5 × 4.8 × 6.8 cm and 5.5 × 3.0 × 4.3 cm, respectively, exhibiting rim enhancement on T1WI (**Figure 1C**) and heterogeneously hyperintense T2WI (**Figure 1D**). She underwent surgical resection of the tumors on October 27, 2021. Precision radiotherapy was administered on December 6, 2021, with the target area including the T2/FLAIR, T1+C abnormal signals, and the surgical cavity. The clinical target volume (CTV) received local radiation therapy extending 2 cm beyond the gross tumor volume (GTV) (DT 46 Gy, 2 Gy/day, 23 days), along with concurrent adjuvant chemotherapy using TMZ (75 mg/m^2^/day). Subsequently, the patient underwent six cycles of adjuvant TMZ chemotherapy (cycle 1: 150 mg/m^2^/day, day 1-day 5, 4 weeks; cycles 2-6: 200 mg/m^2^/day, day 1-day 5, every 4 weeks). Due to residual tumor suspected on imaging, an additional 7 sessions of radiation therapy (DT 60 Gy) were administered on January 5, 2022. The patient’s TMZ chemotherapy was discontinued in June 2022 after 6 cycles chemotherapy. On August 31, 2022, imaging examination revealed progression of the tumor in the left temporal lobe. Chemotherapy combined with targeted therapy was initiated, consisting of bevacizumab (300 mg, day 0) and erlotinib (220 mg, day 1). On April 23, 2023, MRI examination showed edema in the lesion area, and a single dose of bevacizumab (300 mg) was administered for treatment. The patient is currently under regular follow-up, with no evidence of tumor recurrence. She developed colonic adenomatous polyps at the age of 46 years with loss of MSH2 and MSH6 expression (**Supplementary Figure 1**).

#### Patient I:1

On February 11, 2012, the proband’s father, aged 69 years, was also detected a mass in the left frontal lobe, manifesting as a headache. He underwent surgical resection of the intracranial tumor. Following two sessions of postoperative radiotherapy (2 Gy/day), the family declined further treatment. The patient experienced tumor recurrence four months after the surgery and passed away two months later. He had rectal carcinoma at the age of 67 years.

### Histopathological findings

Histological analysis showed that the primary tumor of II:1 was composed of giant and multinucleated bizarre cells, intermixed with smaller and mononuclear cells (**Figure 1E**), with observed “pseudopalisading” necrosis and vascular proliferation. The GBM of II:1r showed diffuse spindle cells arranged in a fascicular pattern with high mitotic activity and scattered pleomorphic cells (**Figure 1F**). The tumor of II:4 was composed of many wreath-shaped multinucleated giant cells and smaller oligodendrocyte-like cells (**Figure 1G**). The tumor of I:1 was also composed of highly pleomorphic neoplastic cells with a predominance of bizarre and multinucleated giant cells (**Figure 1H**). Large areas of necrosis and pathologic vascular proliferation were observed. All three patients were diagnosed with GBM. IHC staining showed positive reactivity for GFAP, Olig-2, p53 (**Supplemental Figure 2**), and S-100, and negative reactivity for IDH1 and Syn. However, the spindle cell component in GBM of II:1r displayed less GFAP and Olig2 expression compared with II:1 primary GBM (**Supplemental Figure 1**). The tumors also showed additional loss of MSH2 (**Figures 1I, K, L**) and MSH6 (**Figure 1J**) expression, with intact MLH1 and PMS2 expression. The proliferative labeling index, detected by antibody MIB-1, ranged from 40% to 50%.

### Molecular genetic landscape of GBMs

**Figure 2:**
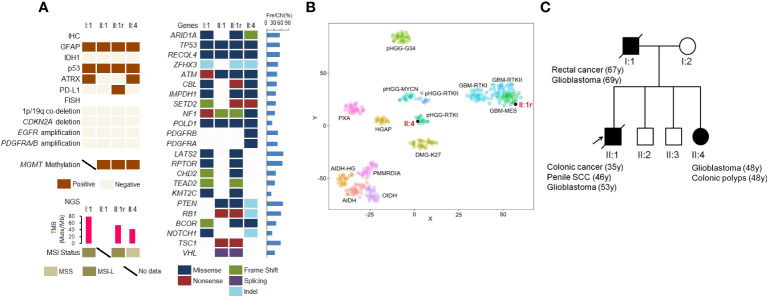
Molecular genetic landscape of GBMs. **(A)** Gene variants in GBMs detected by IHC staining, FISH, and NGS. **(B)** t−distributed stochastic neighbor embedding (t−SNE) analysis of the recurrent GBM of II:1 and primary GBM of II:4. **(C)** The pedigree of Lynch syndrome, showing the familial clustering of cancers. SCC, squamous cell carcinoma. Arrows indicate the probands.

*MGMT* promoter hypermethylation was observed in GBMs from subjects II:1 and II:4. However, common genetic alterations including telomerase reverse transcriptase (TERT) promoter mutation, *CDKN2A* deletion, *EGFR* and *PDGFRA* amplification, as well as chromosome 7 gain and 10 loss, were not identified in any of the three GBMs (**Figure 2A**).

To analyze somatic genetic variations in GBMs, we conducted NGS panel sequencing. As shown in **Figure 2A**, all GBMs in three patients harbored eleven somatic gene mutations, including *ARID1A*, *TP53*, *ATM*, *SETD2*, *NF1*, and *POLD1*. Additionally, *PTEN* and *RB1* mutations were found in II:1 and II:4 and *PDGFRB* and *PDGFRA* missense mutations were found in II:4. MSI testing using NGS showed MSI-L (low) in I:1 and II:1r, and microsatellite-stable (MSS) in II:4. Furthermore, the TMB in I:1 (78.87 Mut/Mb), II:1r (754.23 Mut/Mb) and II:4 (42.25 Mut/Mb) was identified as TMB-H, based on the cut-off of 10 Mut/Mb.

Based on DNA methylation status, GBMs in II:1r and II:4 were classified as GBM-mesenchymal (MES) and high grade glioma (HGG)-receptor tyrosine kinase type I (RTKI), respectively (**Figure 2B**).

We identified a novel heterozygous germline 492 bp deletion in the *MSH2* gene (chr2:47656751-47657242) using WES. According to the variant interpretation guidelines of the American College of Medical Genetics and Genomics (ACMG), this variant is confirmed to be a pathogenic mutation. Furthermore, with familial clustering of cancers (**Figure 2C**), both the clinical phenotype and genotype indicated for the diagnosis of LS.

### Immune microenvironment analysis in GBMs

**Figure 3:**
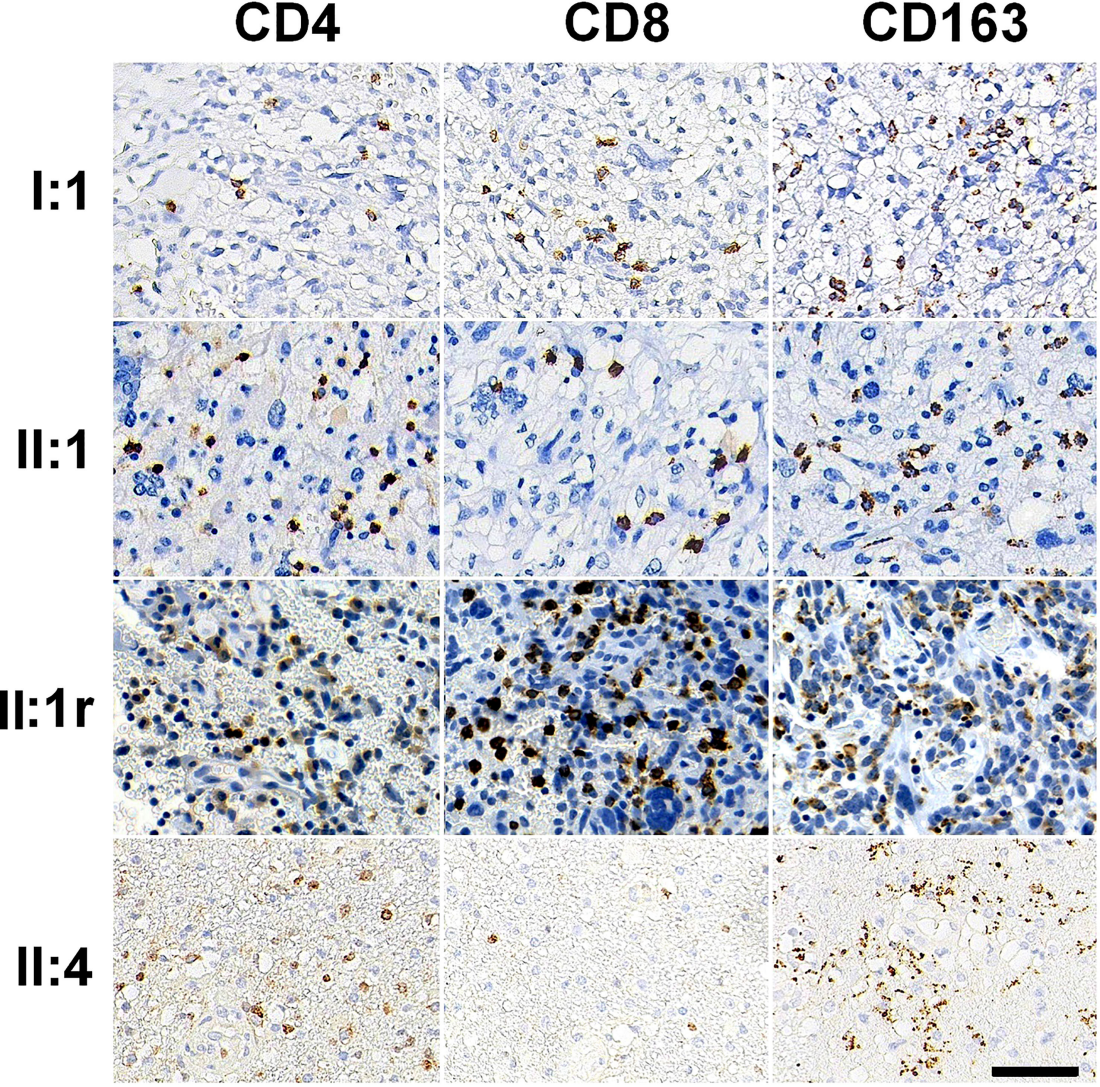
Immune cell subsets in tumor immune microenvironment immunostained by CD4, CD8, and CD163 antibodies in GBM tissues. Bar = 50 μm.

To assess the immune microenvironment of GBMs, we performed immunostaining of tissue sections for lymphocytes and macrophages. We observed few scanty CD4+ T cells in all four GBMs, with the highest density observed in the tumor of II:4 (159.63 ± 107.13/mm^2^). The numbers of CD8+ T cells were higher than CD4+ T cells in each GBM tissue, with the highest densities observed in the tumors of II:1 (440.37 ± 192.39/mm^2^) and II:1r (504.44 ± 263.05/mm^2^). Additionally, macrophages were highly enriched throughout the tumors in all four GBMs, as shown by CD163 immunostaining. Representative photomicrographs of lymphocytes and macrophages infiltration are shown in **Figure 3**, and the densities of immune cell subsets are listed in **Supplemental Table 2**.

**Figure 4:**
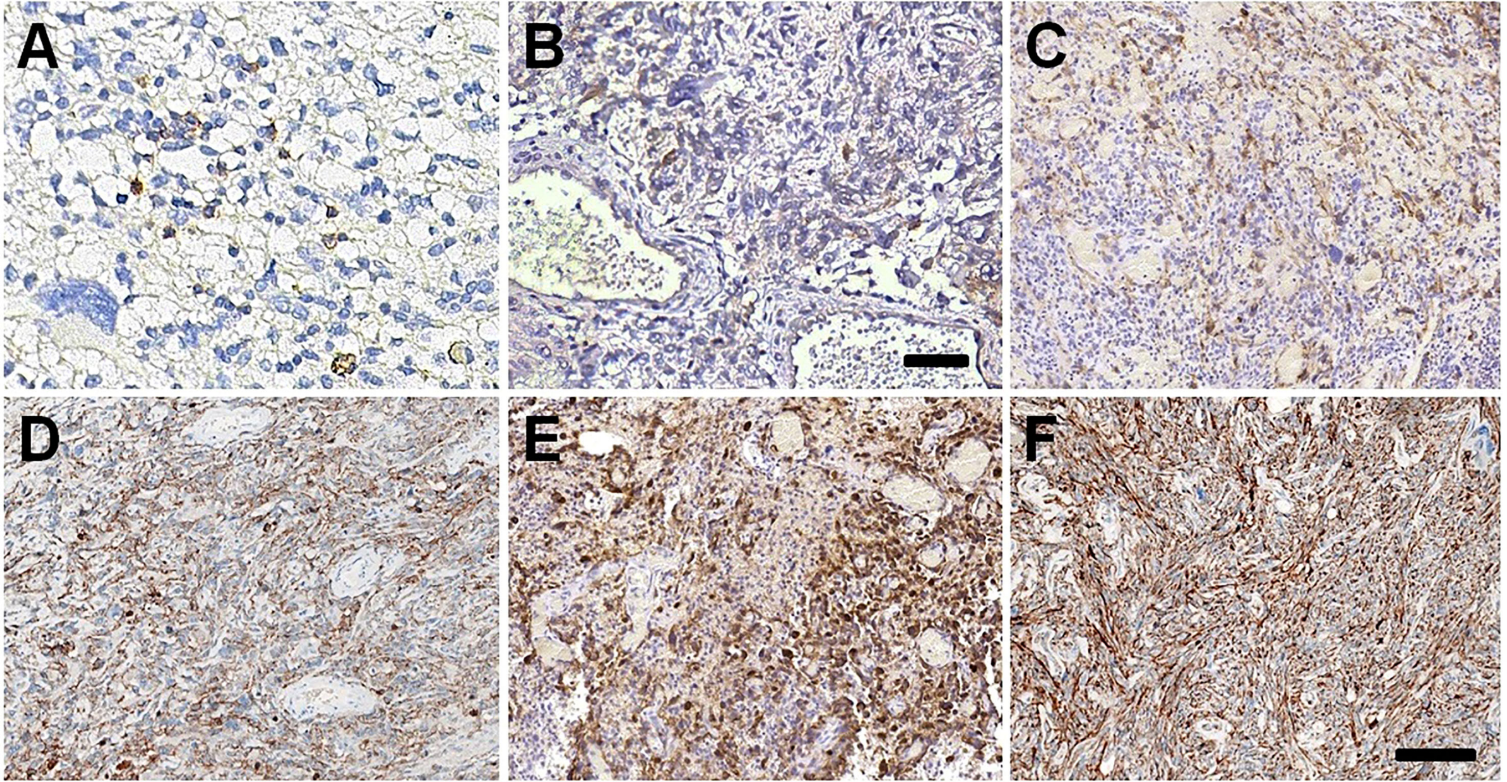
PD-1/PD-L1 expressions in GBM tissues. Few scanty PD-1 positive immune cells in GBM tissues of I:1 **(A)** and II:4 **(B)**. The high expression of PD-L1 22C3 in GBM tissues of II:1 **(C)** and II:1r **(D)**. The slightly stronger staining intensity of PD-L1 SP263 in GBM tissues of II:1 **(E)** and II:1r **(F)**. Bar = 100 μm.

We also evaluated the expression of PD-1 and PD-L1 in GBM tissues. In all GBM tissues, we observed few scanty PD-1+ cells, which were favored macrophages and lymphocytes morphologically (**Figures 4A, B**, **Supplemental Table 2**). Using IHC with 22C3 and SP263 clones, we determined whether GBM tumor cells expressed PD-L1 or not. The TPS of < 1% by both clones in I:1 and II:4 was considered as low PD-L1 expression. II:1 and II:1r had TPS of 75% (**Figures 4C, D**) by the 22C3 assay and 75% (**Figure 4E**) and 80% (**Figure 4F**) by the SP263 assay (**Supplemental Table 2**), indicating high PD-L1 expression. These results firstly confirmed the concordance between the 22C3 and SP263 assays in the primary and recurrent GBMs of II:1.

### Skin lesions on family members

**Figure 5:**
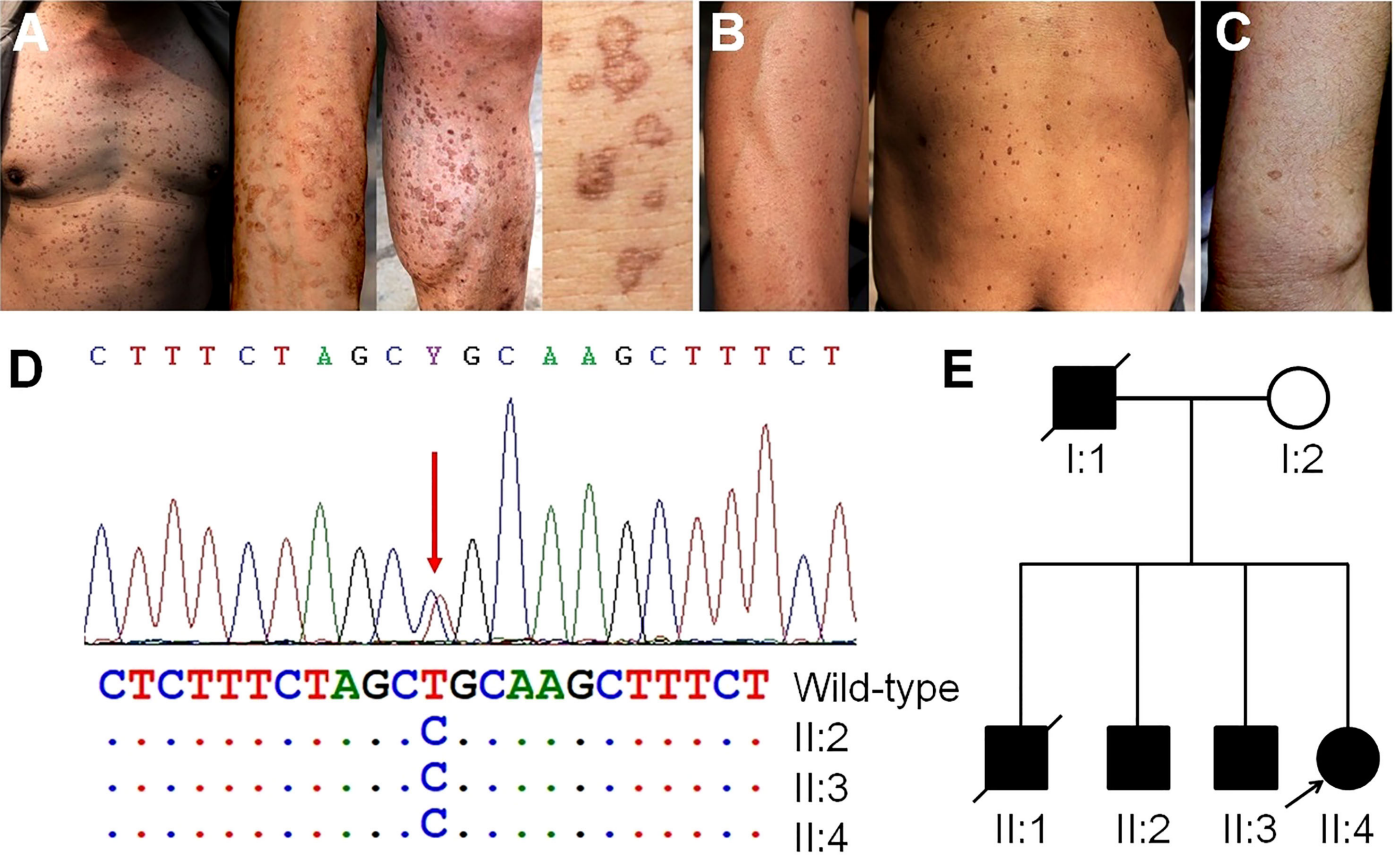
Skin manifestations on family members, germline genetic testing and pedigree investigation. **(A)** Many notable superficial, annular and brownish macules on the trunk, arms and legs of II:2, resulting from occupational exposure to ultraviolet radiation. **(B)** Moderate amounts of macules on the arms and trunk of II:3. **(C)** A fewer macules on the arms of II:4. **(D)** Mutation of the *FDPS* gene in II:2, II:3, and II:4 confirmed by Sanger sequencing. **(E)** The pedigree of porokeratosis. Arrows indicate the probands.

We observed multiple family members with skin lesions characterized by many superficial, annular and brownish macules on their faces, necks, limbs and trunks occurring after the age of 30. The skin lesion on II:2 was notable due to excessive sunlight exposure as a farmer (**Figure 5A**). II:3 (**Figure 5B**) and II:4 (**Figure 5C**) showed fewer macules on their trunks and limbs. A skin biopsy confirmed the diagnosis of disseminated superficial actinic porokeratosis (DSAP). Genome-wide investigation of blood samples using WES revealed a novel heterozygous germline mutation of c.284T > C in the farnesyl diphosphate synthase (FDPS) gene. We confirmed this mutation in family members through Sanger sequencing (**Figure 5D**). The mutation induced aberrant splicing events predicted by MutationTaster (https://www.mutationtaster.org/), and PolyPhen-2 (http://genetics.bwh.harvard.edu/pph2/) predicted it as a possible pathogenic variant with a score of 0.933, resulting in a nonfunctional protein product. We have presented the family pedigree of DSAP in **Figure 5E**.

## Discussion

Primary brain tumors are rare in Lynch syndrome (LS), with a lifetime risk estimated to be between of 1% to 6% (8, 9). However, individuals with LS have a four-fold increased risk of developing brain tumors, mostly high-grade gliomas (8). In a study of 288 families with LS, 14% of the families had a history of brain tumors, of which 68% had mutations in *MSH2* gene, and 56% was GBM (10). Identification of LS-associated GBM patients will lead to further diagnoses through cascade screening, testing and cancer surveillance of at-risk family members. Strategy and pipeline for identifying LS-associated GBM include the following: [1] Personal and family history. In this study, the family history (**Figure 2C**) met the Amsterdam criteria, Bethesda, and revised Bethesda guidelines, which are traditional clinical screening methods to identify individuals at risk for LS who should undergo tumor MMR testing (11). [2] Histological characteristics of GBM. LS-associated GBMs, like the three GBMs in this study, microscopically showed predominance bizarre and multinucleated giant cells (3). However, giant and multinucleated bizarre cells were also observed in NF1-associated GBM (12, 13). Therefore, GBMs with bizarre and multinucleated giant cells require IHC staining for the four MMR proteins. [3] IHC testing to evaluate the MMR status. In this study, the GBMs in three members had IHC loss of MSH2 and MSH6. However, somatic loss of MMR protein was also found in 4.2% of gliomas, including recurrent and TMZ-treatment gliomas (14). Therefore, germline MMR testing was performed subsequently. [4] Germline MMR testing. It is considered the gold standard for LS diagnosis. In this family, germline deletion of exon 7 in the *MSH2* gene caused the formation of a truncated MSH2 protein, resulting in a diagnosis of LS.

We found frequent mutations in *ARID1A*, *TP53*, *ATM*, *SETD2*, and *NF1* in four GBMs, consistent with the genetic analysis of Kim, et al. (15) and Cho, et al. (16). This suggests that disordered chromatin remodeling and a deficient DNA damage response may be implicated in GBM pathogenesis. Notably, the GBMs of II:1 and II:1r exhibited *NF1* and *PTEN* mutations, combined with abundant spindle cells and DNA methylation analysis indicating mesenchymal characteristics. This is consistent with previous studies indicating that mesenchymal GBMs exhibit heightened expression of proinflammatory mediators, immunosuppressive factors, and immune checkpoints. This includes significant infiltration of CD8+ T cells and CD163+ macrophages, as well as high expression of PD-L1 (17, 18), which indicate a suppressive tumor immune microenvironment (TIME). The other GBMs from I:1 and II:4 also showed a significant infiltration of CD8+ T cells and CD163+ macrophages in the tumor immune microenvironment. Previous studies have reported a trend towards increased numbers of CD8+ T cells in glioblastomas with deficient MMR (dMMR) (19, 20), suggesting the potential to convert a “cold” TIME into a “hot” phenotype, which may enhance the effectiveness of immunotherapies. GBM is highly infiltrated by CD163+ macrophages, also known as M2 macrophages, which contribute to the suppressive TIME. These macrophages can promote tumorigenesis and progression through various mechanisms, such as sustaining genetic instability, promoting epithelial-to-mesenchymal transition, and inhibiting adaptive immunity via the expression of immune checkpoint ligands (21). Therefore, reprogramming tumor-associated macrophages to an antitumor M1 phenotype is considered the most promising treatment strategy for GBM (18).

It has been proposed that high TMB is associated with favorable outcomes following anti-PD-1/PD-L1 immunotherapy. In our study, we found TMB-H in three GBMs. Indeed, previous studies have shown that complete loss of MMR protein expression is correlated with increased TMB in GBM samples (15, 16, 20, 22). However, three dMMR GBMs with TMB-H in our study did not show MSI-H status, consistent with recent findings by Indraccolo et al. (20), which suggests that the accordance between MSI and dMMR/TMB may differ depending on the tumor type. In addition, despite TMB-H being detected in GBMs of I:1 and II:4, PD-L1 expression was negative. Hodges et al. (22) reported that GBMs with high and moderate TMB did not have an increased influx of CD8+ T cells, PD-1+ T cells, or tumor-expressed PD-L1, indicating that TMB may not be associated with elevated CD8+ T cells or PD-1/PD-L1 expression. Moreover, the relationship between PD-L1 expression and responsiveness to anti-PD1 therapy is still debated (23). Nevertheless, in a recent study, treatment with PD-1 inhibitor nivolumab resulted in significant clinical and radiographic responses in two siblings with recurrent multifocal biallelic MMR-deficiency GBM with hypermutant profiles (24). Accordingly, in this study, despite the absence of immune therapy in the clinical treatment of the three patients, MMR deficiency and TMB-H may act as predictive factors for an effective response to immune checkpoint inhibitors (ICIs) therapy in GBMs. Indeed, the effectiveness of predictive biomarkers for immunotherapy response needs to be further evaluated in future cases of dMMR GBM treated with immunotherapy.

In this family, we identified the concurrent DSAP, a type of porokeratosis characterized by autosomal dominant inheritance and known to be directly influenced by sun exposure. It typically manifests in the third or fourth decade of life (2). Previously research by Takata et al. described a family with both LS and disseminated superficial porokeratosis but no data regarding germline testing were provided (25). Another study by Lee et al. reported a case of temozolomide (TMZ)-induced DSAP in a patient with GBM (26). However, no DSAP-related germline genetic testing was conducted in that case. In our study, it is worth noting that patients II:1 and II:4 were also undergoing TMZ therapy for GBM treatment. However, DSAP was already present in these patients prior to the development of GBM. Therefore, the occurrence of DSAP in these two GBM patients is not associated with the use of TMZ. It is important to mention that a single-center retrospective study demonstrated a malignant transformation rate of 29.3% in DSAP cases (27). As a result, long-term surveillance is recommended for the family members in our study.

The relationship between DSAP and LS-associated GBM remains unclear. Pathogenic variants in the *FDPS* gene impair the catalysis of farnesyl pyrophosphate (FPP) biosynthesis in the mevalonate pathway. This pathway is known to be critical for cholesterol synthesis to maintain GBM cell growth, including the preservation of GBM stemness and the prevention of apoptosis (28). However, additional studies are required to fully understand the specific involvement of *FDPS* in the development of GBM.

In summary, LS-associated GBM exhibit heterogeneity in clinicopathologic and molecular genetic features, as well as a suppressive TIME. The presence of MMR deficiency and TMB-H may serve as predictive factors for the response to ICIs therapy in GBMs. In addition, the simultaneous presence of DSAP in our patients reminds clinicians and pathologists to consider the possibility of concurrent genetic syndromes in an individual or family, even though many BTPSs are usually accompanied by cutaneous manifestations. Furthermore, this study highlights the importance of identifying the clinical, pathological, and molecular characteristics of LS-associated GBM to benefit patients and family members for diagnosis, genetic counseling, and screening or surveillance protocols.

## Data availability statement

The data analyzed in this study is subject to the following licenses/restrictions: Data are not publicly available due to patient privacy concerns, but can be provided upon reasonable request from the corresponding author. Requests to access these datasets should be directed to Gui-Hui Zhang, 1748@sdhospital.com.cn.

## Ethics statement

The studies involving human participants were reviewed and approved by Institutional Review Board of the Shandong Provincial Hospital Affiliated to Shandong First Medical University (NO. 2023-317). The patients/participants provided their written informed consent to participate in this study.

## Author contributions

Z-GY and FH wrote the original manuscript. Z-GY, FH and G-HZ design the study. Y-JX, Z-HY, Y-CN, X-KL, Z-MZ and M-QZ coordinated patient recruitment and sample collection. Y-HH performed the methylation analysis. Y-HH, X-HG and CM contributed to the genetic testing and data interpretation. ZW and G-HZ took and interpreted histological images as well as made the final pathological diagnosis. ZW, G-CL and G-HZ critically revised the manuscript. All authors contributed to the article and approved the submitted version.

## Supplementary material

Supplementary Figure 1 The metachronous colonic adenocarcinoma and penile squamous cell carcinoma (SCC) of II:1, and colonic adenomatous polyps of II:4. All tumors showed loss of MSH2 and MSH6 expression and intact MLH1 and PMS2 expression.

Supplementary Figure 2 The IHC staining in GBM tissues of II:1 and II:1r. The spindle cell component in recurrent GBM (II:1r) displayed less GFAP and Olig2 expression.
