# Supplementary material for: Characteristics of glioblastomas and immune microenvironment in a Chinese family with Lynch syndrome and concurrent porokeratosis
Source: Front Oncol. 2023 Jul 17;13:1194232. doi: 10.3389/fonc.2023.1194232 (PMC10388537; doi:10.3389/fonc.2023.1194232)
Supplement: Supplementary file 1 [file Table_1.docx]

**Supplemental Table 1.**

Details of antibodies used in the study.

| Antibody against | Clone | Dilution | Ventor |
| --- | --- | --- | --- |
| GFAP | UMAB129 | 1:200 | ZSGB-BIO |
| Olig2 | EP112 | 1:100 | ZSGB-BIO |
| p53 | DO-7 | 1:200 | ZSGB-BIO |
| ATRX | MAX071 | 1:200 | MAXB |
| S-100 | 4C4.9 | 1:400 | MAXB |
| Syn | SP11 | 1:500 | MAXB |
| Ki-67 | MIB-1 | 1:200 | ZSGB-BIO |
| MLH1 | ES05 | 1:200 | ZSGB-BIO |
| PMS2 | EP51 | 1:200 | ZSGB-BIO |
| MSH2 | RED2 | 1:100 | ZSGB-BIO |
| MSH6 | EP49 | 1:100 | ZSGB-BIO |
| CD4 | EP204 | 1:100 | ZSGB-BIO |
| CD8 | SP16 | 1:200 | ZSGB-BIO |
| CD163 | 10D6 | 1:100 | ZSGB-BIO |
| PD-1 | OTI4F10 | 1:100 | ZSGB-BIO |
| PD-L1 | 22C3 | 1:50 | DAKO |
| PD-L1 | SP263 | 1.25 μg/mL | Ventana |
