# Supplementary material for: Characteristics of glioblastomas and immune microenvironment in a Chinese family with Lynch syndrome and concurrent porokeratosis
Source: Front Oncol. 2023 Jul 17;13:1194232. doi: 10.3389/fonc.2023.1194232 (PMC10388537; doi:10.3389/fonc.2023.1194232)
Supplement: Supplementary file 2 [file Table_2.docx]

**Supplemental Table 2.** Quantitative assessment of immune cell subsets and PD-1/PD-L1 expression in tumor immune microenvironment.

| Case | CD4+ T cells* | CD8+ T cells* | CD163+ macrophages* | PD-1* | PD-L1# (22C3) | PD-L1# (SP263) |
| --- | --- | --- | --- | --- | --- | --- |
| I:1 | 46.79 ± 38.33 | 163.57 ± 95.54 | 435.26 ± 95.54 | 23.21 ± 26.57 | ＜ 1% | ＜ 1% |
| II:1 | 31.85 ± 27.76 | 321.48 ± 145.47 | 328.89 ± 132.09 | 22.22 ± 24.25 | 75% | 75% |
| II:1r | 43.33 ± 30.53 | 504.44 ± 263.05 | 537.78 ± 173.31 | 26.67 ± 26.50 | 75% | 80% |
| II:4 | 159.63 ± 107.13 | 110.74 ± 73.93 | 385.93 ± 126.12 | 18.52 ± 16.73 | ＜ 1% | ＜ 1% |

*The results expressed as the number of positive staining cells per mm^2^ (mean ± standard deviation/mm^2^). The positive staining cell counts were made from 50 separate high power fields (400×).

# The PD-L1 tumor proportion score (TPS) was calculated as the percentage of at least 100 viable tumor cells with complete or partial membrane staining.
