# Supplementary figures and images for: Characteristics of glioblastomas and immune microenvironment in a Chinese family with Lynch syndrome and concurrent porokeratosis
Source: Front Oncol. 2023 Jul 17;13:1194232. doi: 10.3389/fonc.2023.1194232 (PMC10388537; doi:10.3389/fonc.2023.1194232)

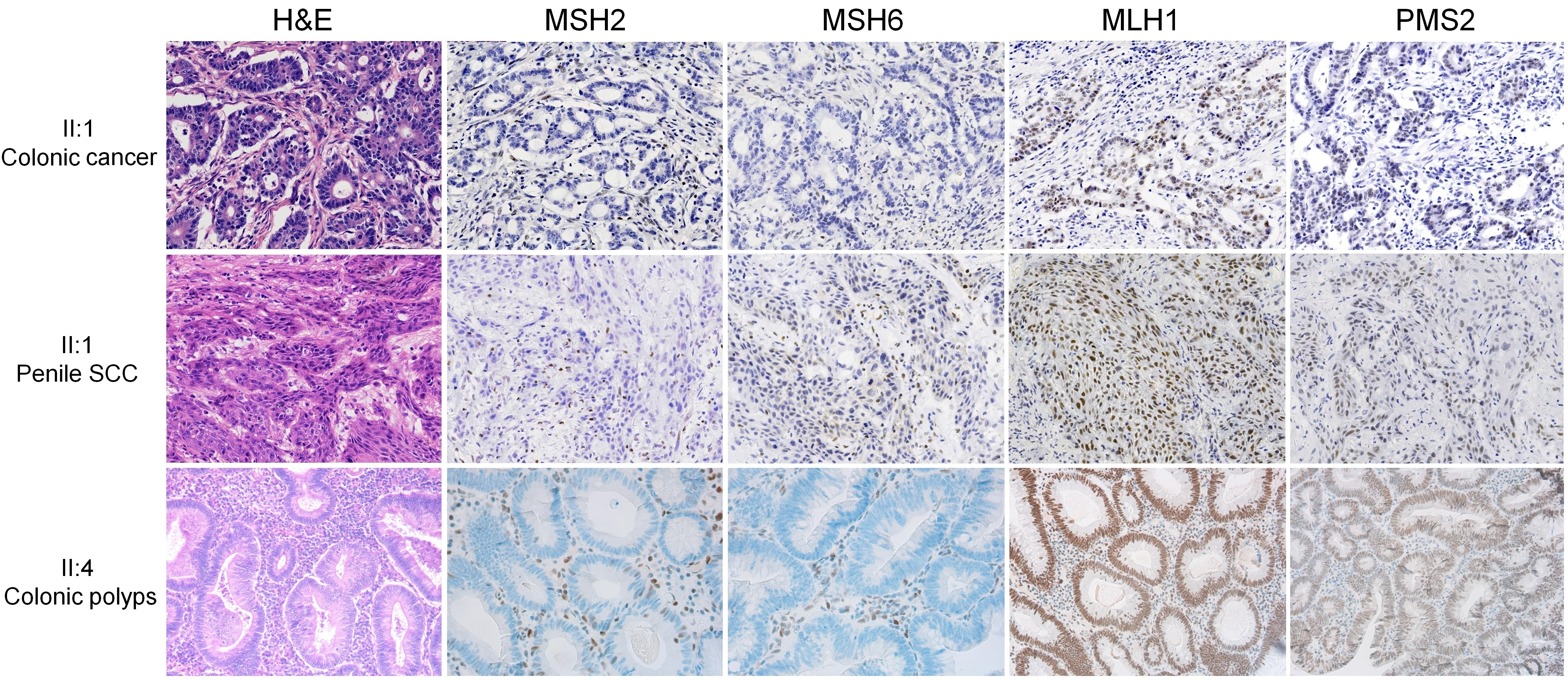

Supplement: Supplementary Figure 1 — The metachronous colonic adenocarcinoma and penile squamous cell carcinoma (SCC) of II:1, and colonic adenomatous polyps of II:4. All tumors showed loss of MSH2 and MSH6 expression and intact MLH1 and PMS2 expression. [file Image_1.jpeg]

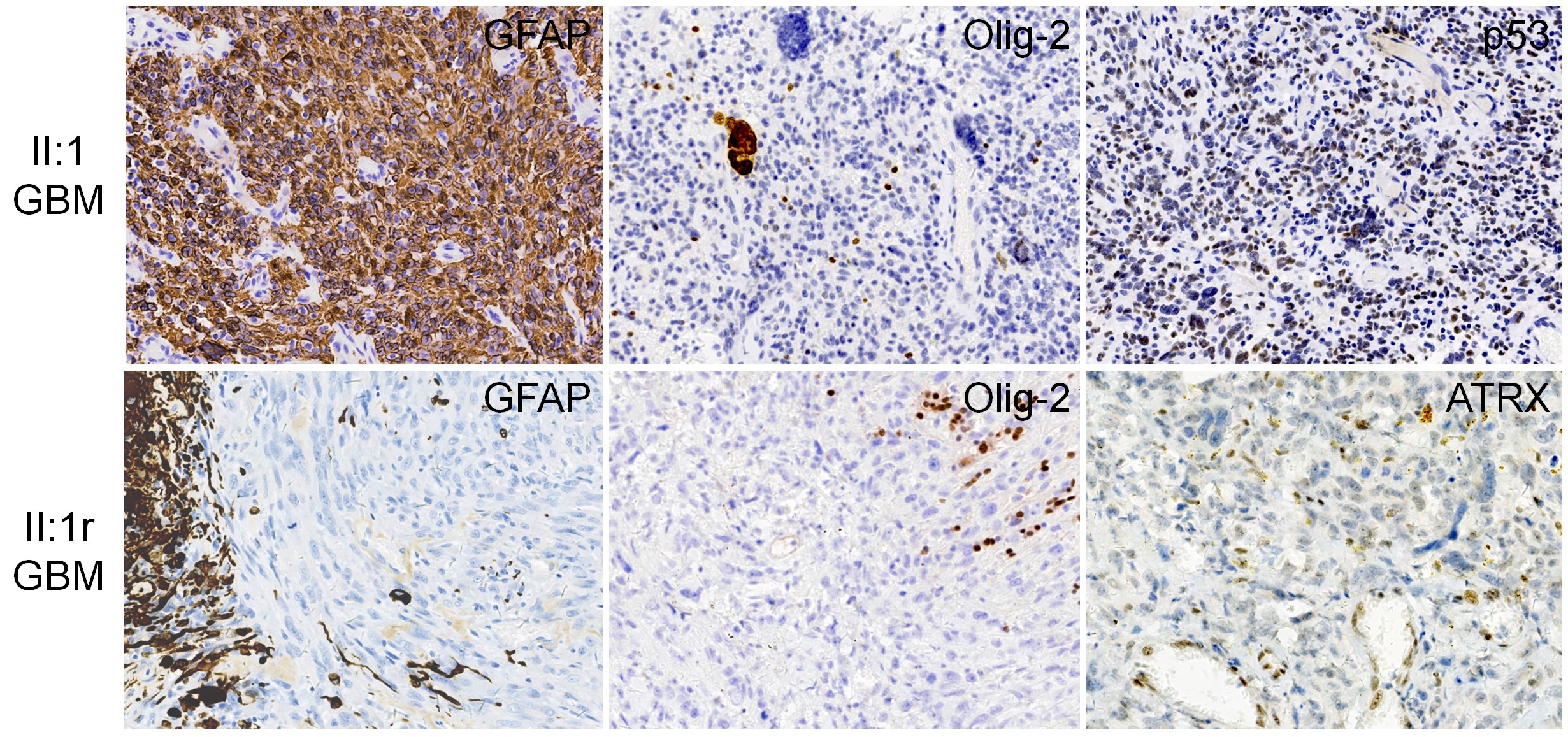

Supplement: Supplementary Figure 2 — The IHC staining in GBM tissues of II:1 and II:1r. The spindle cell component in recurrent GBM (II:1r) displayed less GFAP and Olig2 expression. [file Image_2.jpeg]
